# Reflective and critical thinking in nursing curriculum

**DOI:** 10.1590/1518-8345.2861.3173

**Published:** 2019-12-05

**Authors:** María Antonia Jiménez-Gómez, Lucila Cárdenas-Becerril, Margarita Betzabé Velásquez-Oyola, Marcela Carrillo-Pineda, Leyvi Yamile Barón-Díaz

**Affiliations:** 1 Universidad Nacional de Colombia, Facultad de Enfermería, Bogotá, Colombia.; 2 Universidad Autónoma del Estado de México, Facultad de Enfermería, Toluca, México.; 3 Universidad Nacional José Faustino Sánchez Carrión, Facultad de Medicina Humana, Huacho, Lima, Peru.; 4 Universidad de Antioquia, Facultad de Enfermería, Medellín, Colombia.; 5 Universidad Nacional de Colombia, Facultad de Medicina, Bogotá, Colombia.

**Keywords:** Thinking, Nursing, Education, Curriculum, Nursing Education, Nursing Curriculum, Pensamento, Enfermagem, Educação, Currículo, Educação em Enfermagem, Curriculo en Enfermagem, Pensamiento, Enfermería, Educación, Currículo, Educación en Enfermería, Currículo en Enfermería

## Abstract

**Objective:**

to evaluate the teaching of transversal competence of the Reflective and Critical Thinking that is fundamental in the decision-making and solution of nursing problems, in degree programs of public and private institutions in the Andean region.

**Method:**

multi-center, cross-sectional, exploratory-descriptive study, with mixed approach in 5 countries.

**Results:**

76 nursing programs participated in the study. The Reflective and Critical Thinking was found as a subject, subject content and didactic strategies. Of the 562 subjects reviewed, this type of thinking is found in 46% of the humanities area and 42% in the area of research and professional discipline. It is important to train teachers to achieve coherence between the pedagogical model approach, teaching strategies and evaluations.

**Conclusion:**

nursing programs in the Andean region contemplate the critical thinking as cognitive and personals skills of communication. They also use real situations analysis, supervised practice, simulation labs and specifically learning based in problems to develop the capacity to solve them, decision-making and develop communication skills, including analysis, synthesis and evaluation.

## Abstract (pt)

**Objetivo:**

avaliar o ensino da competência transversal do pensamento crítico-reflexivo, fundamental na tomada de decisões e solução de problemas de enfermagem, nos programas de graduação de instituições públicas e privadas da região Andina.

**Método:**

estudo multicêntrico, transversal, exploratório-descritivo, com abordagem mista em cinco países.

**Resultados:**

76 programas de Enfermagem participaram do estudo. O pensamento crítico-reflexivo foi constatado como disciplina, conteúdo de disciplina e estratégias didáticas. Das 562 disciplinas revisadas, este tipo de pensamento se encontra em 46% da área de humanidades e 42% na área de pesquisa e profissional-disciplinar. Existe a necessidade de capacitar os docentes para obter coerência entre a proposta do modelo pedagógico, as estratégias didáticas e a avaliação.

**Conclusão:**

os programas de enfermagem da região Andina contemplam o pensamento crítico como habilidades cognitivas, de comunicação e pessoais. Da mesma forma, utilizam a análise de situações reais, estágio supervisionado, laboratórios de simulação e, principalmente, a aprendizagem baseada em problemas, com a finalidade de desenvolver a capacidade para solucionar problemas, tomar decisões e desenvolver habilidades comunicativas, incluindo análise, síntese e avaliação.

## Abstract (es)

**Objetivo:**

evaluar la enseñanza de la competencia transversal del Pensamiento Reflexivo y Crítico, fundamental en la toma de decisiones y en la solución de problemas de enfermería, en los programas de grado de instituciones públicas y privadas de la región Andina.

**Método:**

estudio multicéntrico, transversal, exploratorio-descriptivo, con abordaje mixto en 5 países.

**Resultados:**

76 programas de Enfermería participaron en el estudio. El Pensamiento Reflexivo y Crítico se encontró como asignatura, contenido de asignatura y estrategias didácticas. De las 562 asignaturas que han sido revisadas, este tipo de pensamiento se encuentra en el 46% del área de humanidades y el 42% en el área de investigación y profesional disciplinar. Está la necesidad de capacitar a los docentes para lograr coherencia entre el planteamiento del modelo pedagógico, las estrategias didácticas y la evaluación.

**Conclusión:**

los programas de enfermería de la región Andina contemplan el pensamiento crítico como habilidades cognitivas, de comunicación y personales. Asimismo, utilizan el análisis de situaciones reales, la práctica supervisada, los laboratorios de simulación y, principalmente, el aprendizaje basado en problemas, con la finalidad de desarrollar la capacidad para solucionar los problemas, tomar decisiones y desarrollar habilidades comunicativas, incluyendo el análisis, la síntesis y la evaluación.

## Introduction

Globalization brought with it changes in all aspects of life: social, political, economic and cultural. Moreover, the nursing profession is evolving, so that it is increasingly moving away from the biomedical model of care, focused on the instrumental, to focus on people’s health care, with primacy of dialogue and agreements between the professional and the person under care. As part of a multiprofessional team, this requires changes in the curricular proposal and, in turn, a qualifying teacher for a new profile of graduate, whereby reflection, self-criticism and professional responsibility are developed^(^ 1 ^)^.

Therefore, it is necessary to work intensely to reduce the dichotomies that are present in nursing programs, namely: between theory-practice; training and the reality of professional practice; and the student as a passive part of the teaching-learning process and the professional who is required, active, proactive, creative, analytical, with contextual perspective, flexible, with logical thinking, able to carry out a permanent and continuous search for information, able to contribute with his profession to the solution of health problems.

The General Conference of the United Nations Educational, Scientific and Cultural Organization (UNESCO), at its 38th session, held in Paris from 3 to 18 November 2015, “Recommendation about Adult Learning and Education” states in one of its objectives the need to develop people’s capacity to think critically and to act with autonomy and a sense of responsibility^(^ 2 ^)^.

Critical thinking (CT) is a process and a learning outcome ^(^ 3 ^-^ 4 ^)^ and the clinical judgment is the result of this process. The development of the clinical judgment (clinical reasoning skills) is one of the most important and challenging tasks of being a nurse. Clinical reasoning precedes clinical judgment and the decision-making that is important in professional and personal life.

In order to achieve professionals with reflective and critical thinking (RACT), it is necessary to make deep changes in the educational dynamics, in the teaching and student roles, in the use of pedagogy and didactics to transmit knowledge, the curricular structure, the strategies of teaching-learning. These changes are expected to be centered on the student, who must actively participate in the learning process in order to achieve greater development of his or her capacities for reasoning, self-learning, self-evaluation, self-management and self-regulation. Likewise, it is expected that teachers to be critical and creative, attending to individual ways of learning, encouraging the development of good thinking in the student^(^ 3 ^)^.

Literature points out that critical thinking is the “essential foundation for education, since it is the basis for adaptation to the individual, social and professional demands of daily life in the 21st century and beyond”^(^ 4 ^)^. The world changes fast and new realities arise, so there is a fundamental need of people to develop capabilities that allow them to respond and adapt themselves to these changes.

Critical thinking is “the process of seeking, obtaining, evaluating, analyzing, synthesizing and conceptualizing information as a guide, to develop self-conscious thinking and the ability to use this information by adding creativity and taking risks”^(^ 4 ^)^.

Authors pointed out one of the first definitions of critical thinking: “knowledge, skills and attitudes”^(^ 5 ^)^ and, since the end of 1980, various strategies for its teaching have been discussed at all school levels^(^ 4 ^)^. On the other hand, authors point out the importance of generating opportunities to develop RACT in students. Therefore, some authors emphasize the importance of developing it in all health situations in favor of the patient^(^ 4 ^,^ 6 ^-^ 7 ^)^. The nursing professional developing RACT will know where, when and how to use their knowledge, skills, values and attitudes.

The motivation for nurse training in the Andean region became evident in the 1960s. In particular, Colombia generated the first degree program in 1958, which was approved in 1961. In the same period, Venezuela, Ecuador, and Peru initiated undergraduate programs; in the case of Bolivia, it happened only until 1970^(^ 8 ^)^.

The 1980s were marked by the rise of postgraduate programs, increased development of research and the generation of knowledge. The 1990s saw a boom in graduate, specialization, masters, and doctoral programs, the latter especially in education. However, there were also more options for the qualification of nursing professionals for the teaching role. On the other hand, the Higher Education Quality Assurance System (*Sistema de Aseguramiento de la Calidad de la Educación Superior*) was implemented, as well as the Accreditation of the programs and the own regulations or nursing law emerged in each country of the region.

The first decade of the 21st century brought the development of the highest level of nursing education, the doctorate, and, with it, the generation of nursing knowledge in the region and its progress towards consolidation as a discipline^(^ 8 ^)^.

Throughout this journey, there was a permanent motivation for balance and congruence between the graduation profiles and the reality of the job, based on the permanent motivation for the adaptation of the curriculum, the teaching-learning strategies, the evaluation processes, and the teacher qualification to respond to this constantly changing context.

The literature^(^ 9 ^-^ 11 ^)^ shows the need to expand the research in the area of education, to achieve greater development of research and to work in education and nursing policies and practices. On the other hand, the latter shows the need to implement and evaluate pedagogical and didactic strategies that help the student to develop a critical judgment, justified decision making, comprehensive memory and communicative competence^(^ 11 ^)^.

For its part, the Ibero-American Network for Research in Nursing Education (*Red Iberoamericana de Investigación en Educación en Enfermería* - RIIEE), in 2011, identified as a research priority, “The development of RACT in nursing students”, within the tree of the problems detected in its research line Higher Education and Nursing. For the development of this research purpose, the Network suggested a multicenter macroproject with the theme “Strategies to develop the RACT in nursing students: situation in Latin America”. In fact, the conceptual paradigm refers to “critical theory and constructivism, since RACT is an analytical, cyclical, broad and systematic process, but not rigid; its analysis and interpretation allows to have elements for decision-making, as well as to make informed choices”^(^ 8 ^-^ 12 ^)^.

The project involves five of the six regions that make up the RIIEE: the Andean region (Bolivia, Colombia, Ecuador, Peru and Venezuela), Brazil, the Southern Cone, Europe, and Mexico and the Caribbean. The investigation is planned in three stages: 1. Diagnose; 2. Planning and implementation of interventions; and 3. Evaluation. The diagnostic stage includes: 1. The state of the art on scientific production in RACT and teaching strategies for its development; 2. Characterization of educational strategies for teaching the RACT collected in nursing literature; 3. To determine the development of the RACT competence in the different nursing curriculum; 4. To identify the educational strategies used by teachers to develop the competence of reflective critical thinking in nursing students; 5. To identify levels of critical thinking in nursing students according to the classification of Paul and Elder (unreflective thinker - master thinker). Objectives 4 and 5 are currently being developed.

The analysis of the “Scientific Production in RACT in Nursing in 1990-2012 in Ibero-America” produced among others the following conclusion: the formation of a critical reader and the investigative process are strategies that help university students to be critical and autonomous and to access more critically to the knowledge of the disciplinary area. For this, teachers are required to make of each moment and situation in the teaching-learning relationship an open forum for reflection, debate, questioning and contrasting of the different perspectives around the area of professional training and society’s problems^(^ 8 ^)^.

After reviewing the literature on the web of science by using the descriptors critical thinking and nursing, education and curriculum, it was not possible to find studies that analyzed the presence of RACT in the curriculum of nursing degree programs. However, it is very striking the motivation to analyze the importance of its development and studies that demonstrate its value, as well as the use of different and combined teaching-learning strategies to achieve the formation of RACT in nursing students.

This study was done with the purpose of to evaluate the teaching of transversal competence of the Reflective and Critical Thinking that is fundamental in the decision-making and solution of nursing problems, in degree programs of public and private institutions in the Andean region.

## Method

The coordinating group of RIIEE developed the research project from which the research groups of each country and region of the Network were made up, integrated by members of the Network and researchers in education and nursing, who are also teachers in Higher Education Institutions (HEI), and mostly with PhD academic level. The HEI in Nursing (HEIN) were identified through the Ministries of Education, the Associations of Schools and Universities of Nursing of each country and the Latin American Association of Schools and Universities of Nursing (*Asociación Latinoamericana de Escuelas y Facultades de Enfermería* - ALADEFE).

The project was benefited from the Declaration of Helsinki (Ethical principles for medical research on human subjects) and the current ethics legislation in each country, was approved by the Ethics Committee of the University of Antioquia, Colombia, by Act No. CEI-FE 2015-25 on July 31, 2015. The respect for privacy and confidentiality were ensured to each Program Director, with the informed consent signed by each participant. The project was also sent to them and their understanding was guaranteed. In turn, we conceded the right to choose what information they wanted to share. Confidentiality was maintained by institutional coding. Finally, was given a deadline of 15 days to obtain the response of acceptance to participate.

The target population of this research were 187 undergraduate nursing programs in the Andean region: Bolivia (47), Colombia (47), Ecuador (21), Peru (62) and Venezuela (10). We considered only the nursing curriculum of the HEI, recognized by the Associations of Schools and Colleges of Nursing of each country or its counterpart, regardless of whether they were public or private. Resulting in the nursing curriculum of 76 Institutions that correspond to the 40.64% HEIN that teach undergraduate nursing in the Andean region: Bolivia (7), Colombia (38), Ecuador (11), Peru (14), and Venezuela (6). Due to the difficulty in obtaining the information, we checked web pages, contacted HEIN members, made contacts by telephone, in some cases, we made personal visits and, finally, the complete program was requested in PDF format for the complete the instrument of the research group. In addition, the HEIN did not refused to participate, but some institutions did not respond to the invitation. The result of this process: 30 institutions accepted to participate and provided the complete information, and out of 46 partial results were obtained. An HEIN database of the names, telephone numbers and e-mail addresses of the authorities in charge of managing the programs was created in order to follow up on their responses.

After identifying, during 2011 and 2012, the theoretical and conceptual framework of RACT from different authors and different perspectives (education, pedagogy, psychology and nursing), despite the abundance of literature about the subject, we concluded that the concept is very unclear from a nursing point of view^(^ 13 ^-^ 14 ^)^.

However, it was necessary to establish a concept that was accepted by the research group of the Network, that allowed to determine a starting point or consensus to carry out this work and, without detriment to seek some level of fidelity to the multiple approaches of the scholars of this research object, that was understandable for the group and reflected what was intended to be done in its research phases and stages.

The Network took as a theoretical framework the approaches made by Paul^(^ 15 ^)^ and Paul; Elder^(^ 16 ^)^, the elements of the CT and the attitudes of the Critical Thinker proposed by these authors. With the material analyzed, RIIEE constructed the following concept: “Reflective and critical thinking is a complex, systematic and deliberate process of reasoning, self-directed and action-oriented. It is primary purpose to choose, based on intellectual and affective processes (cognitive, experiential and intuitive), the best response options that favor the solution of nursing problems, in well-defined contexts and in accordance with the ethical postulates of the profession that allow it to act with rationality and professional autonomy”^(^ 8 ^)^.

The research process included the conceptual and theoretical analysis of the curriculum, the updating of the context of research development in nursing education in each country of the region, the characterization of the HEIN and, finally, the results of the state of the art on teaching strategies for the development of the RACT 1990-2012, Andean region.

Once the exhaustive bibliographic review was carried out, the instrument was designed based on the concept of Stenhouse^(^ 17 ^)^, the curriculum as a macro concept that encompasses the socializing function of the school is at the same time pedagogical ideas, structure of contents in a particular form, precision of them, reflection of educational aspirations more difficult to translate in concrete terms and skills to promote in the students^(^ 16 ^)^. In Posner^(^ 18 ^)^, who raised the great number of phenomena involved in the curriculum; Gimeno-Sacristán; Pérez-Gómez^(^ 19 ^)^, there is five categories in which the definitions of curriculum can be articulated: as an organized knowledge structure, production technology system, instructional plan, set of learning experiences and problems solution.

Based on the aforementioned, the specific instrument for this investigation was constructed with three parts. The first with 10 items, with general information from the HEI or University. Each University is subdivided into Centers that are parted into Colleges and these are divided into programs: name, geographic location, type of institution, accreditation data, mission, vision, objectives, curricular guidelines for degree programs, web page, and data about who completed the instrument. The second, 28 items, for the College, School, Department or Nursing Program (typology to identify HEIN in the region), with the following subsections: general aspects of the nursing program, character within the institution, number of sites where the program is offered, accreditation data (date, resolution, and time of accreditation), program justification, mission, vision and objectives of the program, evaluation process, graduation profile, pedagogical model, number of hours and credits, curricular structure (nursing program subjects that correspond to each area or component). Finally, the general characteristics of the teachers: kind of affiliation with the institution, time worked, and maximum educational level achieved. The third, 9 items, for specific information about each of the subjects: name, component or area to which it belongs, number of hours and credits, type of subject (theoretical, practical and theoretical-practical), contents, teaching-learning methodologies and evaluation process.

The members of the research group carried out an analysis of the validity of the content of the instrument. Afterwards, the pilot test was conducted, starting with its implementation in each of the HEI in which the researchers worked; the results were analyzed and the corresponding adjustments were made in its structure. Subsequently, the adjusted instrument was tested with five members from the region, one from each country, but different from the research group. Because of this test, we decided to design a guide to facilitate the completion of the instrument and ensure objectivity in the collection of information, because of the language differences. It is possible to obtain the final version of the instrument from the authors of the project.

Each participant received the letter of invitation, the project, the informed consent, the instrument to collect the information and the corresponding guide for its completion in hands and by e-mail.

The information obtained was reviewed and, in some cases, it was necessary to request the complementation of some aspects of the instrument. Then we proceeded to codify the HEI or Universities and the HEIN. The information was included in Excel tables designed with the predetermined categories and subcategories, which were later incorporated into the SPSS statistical analysis program, version 19. The information was processed using descriptive statistics, with frequency distributions and average analysis, and analyzed by institution, by country and as an Andean region, according to the categories and subcategories determined, allowing comparisons between countries and conclusions to be drawn as a region.

The analysis of the information was carried out using the deductive-inductive method, considering the objectives of the project and the revised conceptual theoretical framework, with the aim of determining the presence of the RACT, explicit and implicit, in each categories, the coherence of the approaches between University-College-Program, the coherence between the objectives, contents, teaching-learning strategies and the evaluation process in each subject. In this sense, we analyzed the linearity or coherence with respect to what was proposed, developed and evaluated in relation to the RACT and, finally, the contradictions and inconsistencies found in the aforementioned approaches were pointed out. We considered national and international studies about the subject for the analysis and discussion of the results obtained, in addition to the documents mentioned above.

## Results

According to the information obtained by the research group, the Andean region has 2,552 HEI; 410 with character of universities and 160 are public, 220 are private and 14 are in special regime. There are 167 universities with nursing programs, 146 affiliated and recognized by the respective Associations of Schools and Colleges of Nursing in each country. The number of accredited nursing programs in the Andean region is 43: Colombia (20), Peru (20) and Bolivia (3). Precisely, of the 20 accredited institutions in Colombia, 11 already have their certifications renewed, which are of 8, 6 and 4 years; 5 and 6 years for Ecuador and 3 years for Peru. Bolivia is just beginning the process and Venezuela has no information about it.

The total population of HEIN by country was Bolivia 47, Colombia 42, Ecuador 21, Peru 62 and Venezuela 10. A total of 76 HEIN answered: Bolivia 7; Colombia 38; Ecuador 11; Peru 14 and Venezuela, 6. These institutions constituted the sample of the study.

Twenty-one of these institutions are certified: in Colombia 20 and in Bolivia 1; 12 did not include this information and 41 were not yet certificated. Of the total number of institutions that provided the information, 47 are public, 26 private and three do not know the information. Administratively, 36 are programs; 22 Colleges; 21 Schools and one Department.

The number of hours and credits of the programs showed considerable heterogeneity: the average number of hours was 5,552.3, corresponding on average to 232.11 credits. Regarding the number of hours per credit, the lowest is in Peru, which has 13 hours per credit, and the highest is in Colombia, with 48 hours corresponding to one credit. There are institutions that do not work with credits, especially in Bolivia; others did not included this information, among them Ecuador and Venezuela.

The main characteristics of the 912 teachers developing nursing programs in the Andean region are: 501 (54.9%) with a Specialist degree; 634 (69.51%) with a Master’s degree and 58 (6.35%) with a PhD; 249 (27.3%) with a postgraduate degree in Education.

From a general perspective, it should be noted that of the five countries in the region only Venezuela and Peru explicitly present the RACT in their Organic Law (OL) or Higher Education Law in terms of integral and permanent formation of reflective critical citizens (LOE, 2009, or Organic Law of Education, in Venezuela)^(^ 20 ^)^ and (Law 30220, 2014, or University Law, in Peru)^(^ 21 ^)^.

The results of RACT’s presence are presented below: Universities or HEI; in Colleges, Programs, Schools and Departments, that is, in HEIN; and in the subjects.

When analyzing the information of the Universities or HEI, we found the RACT as direct mention, indirect mention and evidence of traditional positions was found. Directly, it was found as a training purpose in Bolivia, Colombia and Peru: *receptor and analytical constructor, with critical conscience;* as methodology to achieve it, in Colombia and Peru: “*promoting reasoning, the CT and creative”;* as a result of learning in Ecuador and Venezuela: *capable of solving problems, CT promoter.*

The indirect mention was found as result in the five countries of the Region, as a strategy in Bolivia, Ecuador, Peru and Venezuela: *integral formation, relation practical theory;* and as objective in Colombia: *future graduates with ethical conscience, autonomy, democratic spirit and highly qualified.*

There are still traditional postures: *teaching, evaluation as a final product, training in instrumental action, the educational process as providing knowledge.*

By going a little deeper into the HEI, we found that 88% (38) consider the RACT: 63% (27) in the mission; 7% (3) in the vision; 51% (22) in the objectives and 30% (13) in the curricular guidelines. Among these, three defining categories were identified. The first, as a training purpose: *prepare professionals and leaders with CT and social conscience*. The second, as a methodological strategy to achieve its development: *to develop and implement pedagogical methods that encourage reasoning, CT and creativity, and that encourage habits of discipline and productive work*. And the third, as a result of the formation process that includes the subject: *Training of critical, self-managed, creative and proactive men and women;* and, moreover, refers to the projection and utility: *with the promotion of CT and the generation of knowledge, thanks to the strengthening of critical analysis, anticipation and vision of the future and development of viable alternatives to the problems.*

At HEIN, RACT is expressed in the graduation profile, objectives, curricular guidelines and mission. Table 1 shows the data summarized in relation to the number and percentage in which the RACT is presented in the subcategories and with regard to the total. The information recovered allows us to identify that the RACT ranks first with 38.3% in the graduation profile, followed by 35% both in the curricular guidelines and in the objectives; thirdly, is in the mission, 26.7%, and finally, with 11.7% it is in the vision. Bolivia has the highest percentage of presence in its curricular guidelines, followed by Colombia in its graduation profile, objectives, and mission, while Venezuela is in one before the last place with a 28% of presence in its mission and is not present in the profile or in the curricular guidelines. Peru has the last place and presents it only in the objectives of the programs.

**Table 1:** Number and percentage of RACT* presence in the categories analyzed in Higher Education Institutions, by total sample and subsamples in the countries of the Andean Region, 2017

Countries	Institutions	Mission		Vision		Objectives		P. Graduation		Curricular guidelines	
		RACT*	% RACT^†^	RACT*	% RACT^†^	RACT*	% RACT^†^	RACT*	% RACT^†^	RACT*	%RACT^†^
Venezuela	7	2	28.6	1	14.3	1	14.3	0	0.0	0	0.0
Peru	15	2	13.3	1	6.7	4	26.7	3	20.0	3	20.0
Ecuador	13	1	7.7	3	23.1	3	23.1	3	23.1	10	76.9
Bolivia	7	3	42.9	2	28.6	3	42.9	2	28.6	7	100.0
Colombia	18	8	44.4	0	0.0	10	55.6	15	83.3	1	5.6
Andean Region	60	16	26.7	7	11.7	21	35.0	23	38.3	21	35.0

* RACT = Reflective and Critical Thinking;

† %RACT = Percentage of Presence of Reflective and Critical Thinking

In a cross-sectional view of what is proposed by curricular programs, three categories were identified to be highlighted. The first, the development of cognitive and personal skills, expressed as *the training of professionals with scientific, technical, critical, analytical and reflective knowledge, as well as communication, oral and written expression skills;* and referred to *a critical, creative, participative, supportive, innovative and sensitive attitude towards social change.*

The second, the way in which its development could be achieved, among which the research stands out: *promote and develop research, generating knowledge in the different areas of nursing that contribute to universal science and the solution of health problems;* and the use of technologies: *learns permanently developing the capacity of abstraction, analysis, synthesis and using information technologies*. The third, its finality, related to *the ability of individuals, families and community groups to interfere and make decisions in the solution of health problems,* to provide *comprehensive care with the capacity to solve health problems in changing and emerging environments.*

Concerning the pedagogical models expressed in the HEIN, a variety was found in the denomination. First of all, the constructivist approaches are highlighted in eight (8) Institutions, with some connotations as the model *social-critical-constructivist* and second, the *cognitive - humanistic* in four (4). Other models or approaches were also identified, among them: dialectic, technological, psychological, the *problematic* schools, the *Active, Reflective, Dialectic, Innovative* and *Critical*. Finally one institution works with the model *based on the pillars of education*, in which learning to know, learning to do, learning to be and learning to live together, which includes, educating for life, educating for life, educating for work, educating in society and for society^(^ 22 ^)^.

*The RACT in the subjects of the programs of Nursing in the Andean region*

Only 29 of the 76 HEIN participants in the study were able to obtain information on subjects (38.15%), and 22 (75.86%) of these in nursing programs, RACT was present in different elements of the subjects. 562 subjects were reviewed, 159 (29%) of which have no information about teaching strategies or evaluation. Moreover, some programs record the same teaching and assessment strategies for all subjects in the program, 45 (8%).

Table 2 presents the results by subcategory and the total presence of RACT in the different groups of subjects, basic area or foundation subjects (which introduce and contextualize the student in the field of knowledge), Research, Humanities (the study of the behavior, conditions and performance of the human being), disciplinary professional area (gives the basic grammar of the profession and discipline) and those of the flexible area (the student chooses them according to personal interests, allow to the learner to approach, contextualize and study in depth aspects of the profession and discipline, allowing to learn tools and other kinds of knowledge, leading to develop interdisciplinarity, flexibility and diversity).

**Table 2:** Number and percentage of PRYC† in areas in which nursing degree programs are divided by total sample and sub-samples in Bolivia, Colombia, Ecuador, and Peru, 2017

Countries	Basic or Foundation			Research			Humanities			Disciplinary or Professional			Flexible Area		
	Sub*	RACT^†^	%^‡^	Sub*	RACT^†^	%^‡^	Sub*	RACT^†^	%^‡^	Sub*	RACT^†^	%^‡^	Sub*	RACT^†^	%^‡^
Peru	21	7	33	12	6	50	2	1	50	53	29	55	1	0	0
Ecuador	56	6	11	13	1	8	27	6	22	96	24	25	6	0	0
Bolivia	17	4	24	7	3	43	6	4	67	64	27	42			
Colombia	50	17	34	9	7	78	20	14	70	94	49	52	8	4	50
Total	144	34	24	41	17	42	55	25	47	307	129	42	15	4	27

* Sub = Subjects;

† RACT = Number of subjects presenting Reflective and Critical Thinking in each area;

‡ % = Percent of subjects that present reflective and critical thinking in each area

The information provided makes it possible to indicate RACT as a subject: *Workshop of Critical Thinking and Introduction to CT*; second, as a subject content: *CT in Nursing,* and, third, RACT is evidenced in teaching-learning strategies.

The highest percentage of subjects in which RACT is evident correspond to the area of humanities, with 46% (55), in which analysis of real situations, group work, concept maps, role playing and seminars are predominant.

In second place, it is in the professional-disciplinary area with 42% (307) subjects with the predominance of the following strategies: supervised clinical practice, clinical case, problem-based learning, simulation laboratories, and the nursing process. The research is in the same place, 42% (41) subjects. The most commonly used strategies are: critical discussions of research reports and articles, project development, workshops, and problem-based learning.

In the last place, subjects from the basic or foundation area 144 (24%). Including discussion workshops, concept maps and case studies.

A great variety of strategies have been identified, among them are: *presentation and discussion of clinical case, group work, clinical practice, flipchart, observation guides, debates, discussion about specific topics, resolution of case studies, support of the nursing care plan, investigative reports , workshop development.*

What is evaluated: *the development of competencies, the acquisition of skills, the development of superior cognitive processes, the professional spirit and the development of processes and independence.*

Finally, in some of the subjects, the intentionality of the evaluation of the RACT is explicitly presented: written works about the topics of each seminar in which the proper handling of the bibliography is evidenced, *the capacity for criticism, analysis and synthesis, evidence of problem solving, case analysis and Nursing Based in Evidences*, didactic relationship analysis and *fundamental elements of the RACT, conceptual knowledge, written and oral reflections, group work, practical reflections and group discussions.*

It is evident that traditional evaluation techniques still exist: evaluating procedural aspects, dexterity, motivation and initiative in the procedures, memory evaluation, participation in class, oral and written interventions and, finally, the replication of the topics studied in classes.

Therefore, the analyzed programs show interest in including as an important element in their future graduates the development of the RACT. This aspect is vanishing in the development of the subjects. It is evident in the pedagogical strategies, but it is lost until disappearing in most of the evaluative processes.

## Discussion

The analysis results of the plans and programs of the HEI and HEIN allow to conclude that the proposes of the Law of Higher Education to develop the RACT in the students does not guarantee that it is included in the subjects and evaluations.

What is stated in HEI and HEIN allows us to infer that epistemological and theoretical contradictions are present in the Institutions and among them. It makes necessary an epistemological, theoretical and methodological consideration in order to achieve alignment and coherence between the purposes in the curricular guidelines and what is programmed in the curricular plans for the concrete work with the students. This matter goes against comprehensive training, since it is demonstrated that critical and reflective skills contribute to train professionals with greater ability to care for patients^(^ 23 ^)^.

It should be noted that it is the University or HEI that determines the philosophical bases that will guide the academic units that compose it, so that they, in turn, incorporate these principles into their academic programs. The results show that there is no linearity between the proposals of the university with respect to its mission, vision, objectives, graduation profile, curricular guidelines, and what is proposed in the nursing degree programs. There is more linearity in Institutions with a longer trajectory and development, private and public ones.

The analysis of the areas in which the subjects are grouped made it possible to identify that the subjects of the humanities area have the highest percentage of presence of the RACT. This result can be explained by the strategies used, but even more by the subjects under study, since it has been demonstrated that the teaching-learning strategies based on the humanities have a significant impact on the development of skills such as clinical reasoning^(^ 24 ^)^. The subjects in the professional area use strategies such as case study, supervised clinical practice and other relatively new ones as problem-based learning and simulation laboratories. Strategies that, by involving simulation or potential practical actions, contribute to enhance critical skills and make decisions that lead to the future professional committing fewer errors during the care of patients^(^ 25 ^-^ 26 ^)^.

By contrast, it is not the same with the subjects of the foundation or basic area in which it is necessary to return to some knowledge aspects that already exists, such as anatomy, physiology, anthropology, psychology, statistics, among many others. For some students these topics are very difficult and involve, on several occasions, an excellent dose of memory. However, the teaching strategies that develop the RACT are not so frequent. It is important doing more research on this point to sustain if it is true.

On the other hand, for the majority of HEIN, training is conceived as qualification and progress achieved by people and as a principle of theories, concepts, methods, models, strategies and courses of pedagogical action that aim to understand and qualify the teaching. In some cases, the transfer of knowledge is approached, but it still underlies the concept of learning as acquisition of knowledge built and finished; the teacher is the one who has the knowledge and the student is who learns what teacher knows.

The curricula of the Andean region include explicit elements that contribute to the development of the RACT, such as reading, writing and reasoning, allowing to the future professional to know how to learn, reason, think creatively, generate and evaluate ideas, make decisions and solve problems^(^ 24 ^)^. It includes as proposals the development of social skills, with emphasis on oral and written communication, cognitive skills including problem solving, establish different alternatives, understand the consequences of actions, make decisions and critical thinking^(^ 16 ^)^. Also, intend to achieve in the student some characteristics of the critical thinker like to be creative, innovative, proactive, analytical, participatory, entrepreneurial, self-critical, supportive, humanistic, ethical and scientific^(^ 27 ^)^.

Regarding the pedagogical models proposed by the HEIN, inconsistencies between the approach of constructivist approach and meaningful learning are evident. The axis is the student and the repetitive approach in the subjects with master class methodology, reading guides and analysis made by teacher, but not by the student. It shows a traditional model centered on the teacher, with an emphasis on memory, comprehension and the application of concepts. Some subjects focus learning on the acquisition of concepts, despite using the integrating project as a teaching-learning strategy, workshops and practice as evaluation. The pretense for the development of the RACT is not in line with the evaluation, with the examination, in the application of contents, since it is centralized in aspects of memory and knowledge, in an asymmetric theory-practice relationship.

Although significant learning is intended and the importance of integrating it into the formation of learning approaches with the intention of promoting critical thinking, added with successful learning experiences^(^ 28 ^)^, it is not really concrete how it could be achieved. Strategies such as simple repetition and teaching for the acquisition of concepts show the persistence of the traditional educational models.

This study found there is no a clear structure to operationalize the theories of the proposed pedagogical models, even though there are expressions that point to RACT. Thus, the elements important for its development are presented in the teaching and learning strategies in a more remarkable way.

The curricular guidelines express the intention to transcend technical rationality and behavioral objectives^(^ 29 ^)^, from the positivist, rationalist or empirical analyst paradigm, to the humanist and critical curriculum^(^ 30 ^)^ to the socio-critical paradigm and critical thinking based on hermeneutic processes^(^ 31 ^)^. The social and contextual (political, economic and cultural) aspects that influence and determine the health behaviors of the people are still incipient in the curricula^(^ 32 ^)^.

According to what has been demonstrated, it is possible to state that there is no predominance of a pedagogical model, but a mixture of several models in the same program with varied influences. The presence of the following models was identified: Traditional Pedagogical, Behavioral, Cognitive, and Social Pedagogical, the latter being very tenuous^(^ 33 ^)^.

There are four fundamental elements to forming critical thinkers: first, the question; second, the creation of continuous opportunities to participate in dialogue, debate, research, and critique; third, self-evaluation and hetero-evaluation; and fourth, teachers as models of critical thinkers^(^ 32 ^)^. Considering these elements, we can assure that the creation of opportunities is present with more intensity in some curricula, and self-evaluation and hetero-evaluation have begun to be implemented especially in public institutions.

Mentioning the subjects, it is not evident that the thought is motivated by complex kind of questions that encourage exploration, generate evaluation, create concepts and knowledge^(^ 33 ^)^.

The literature points out that the Socratic questions stimulate the student to use existing knowledge, since they promote a greater understanding and integration of new knowledge, they foment the habit of thinking critically^(^ 8 ^,^ 34 ^)^. Other authors suggest, for the reports, questions about the purpose, information, concepts, assumptions, implications, points of view and the questions, as elements that favor analysis, the evaluation of ideas and reasoning^(^ 24 ^,^ 35 ^)^.

Like other researches, this study found that the most used strategies in the progress of the professional area that promote the development of RACT are the case study^(^ 24 ^,^ 36 ^)^, problem-based learning^(^ 24 ^)^, supervised clinical practice^(^ 37 ^)^, the nursing process^(^ 4 ^,^ 38 ^)^ and simulation laboratories^(^ 34 ^,^ 37 ^-^ 38 ^)^. In this article, we only refer to two of these strategies, which were selected because of the great advance of information and communications technologies. The growing need to access this kind of infrastructure as a fundamental part in the training of future professionals and as an example of a single teaching and learning strategy is not sufficient to achieve the RACT, rather, the use of different techniques enhance its development, as we will see below.

We agree with the conclusion of authors who suggest that Problem-Based Learning and simulation labs are active strategies that develop RACT in nursing students^(^ 37 ^)^.

The case study, moreover, promotes active learning, helps to solve clinical problems, promotes the development of critical thinking skills^(^ 34 ^-^ 35 ^)^, in addition, it allows to integrate knowledge, to think as a professional, to analyze individual situations in specific contexts from different angles, to use theoretical concepts in the delimitation of a concrete problem^(^ 36 ^)^. It also stimulates collaborative and team work, the work with different points of view. The question-problem is the motivator in the search for alternative solutions, is useful in simple and complex situations, allows to apply theory in practice, promotes the exchange of ideas, teaches students to learn to control their own thinking and promote the exchange of ideas and intellect^(^ 37 ^)^. In addition, it helps to incorporate time management and take responsibility. It also facilitates the integration of the four elements of the Nursing metaparadigm: the person receiving the care, health as purpose, the nature of the nursing and the context or environment.

The case study allows the simultaneous implementation of other strategies that further enhance the development of RACT, such as concept maps, the analysis and selection of scientific evidence, the nursing process, nursing history, role-playing, argued discussion and debate.

In contradiction to all the positive aspects of the case study in the development of RACT, the dichotomy between theory and practice in a large number of the curricula reviewed is an obstacle to achieving all the benefits pointed out. Since some teachers are in charge of the development of the theoretical subject in the classroom, others are in charge of their practical part in other spaces that require this care.

Regarding the practice based on simulation models, a study^(^ 38 ^)^ shows how the promotion of RACT is relevant. In this connection, it highlights the importance of including simulation as a key element in curricula, because it ensures skills in this kind of thinking^(^ 38 ^)^ and gives students the opportunity to show their ability in decision-making, critical thinking and other skills^(^ 39 ^)^. Other authors emphasize its importance when students reflect it on their thinking process and show how it guided their actions^(^ 34 ^)^.

There is efficiency of simulation laboratories when accompanied by active strategies, such as the conceptual map before each laboratory session, a visual aid that allows the concepts, objectives, justification, expected results and possible complications to be described in a logical manner if the procedure is not carried out in the appropriate manner^(^ 34 ^)^. The same author suggests the use of high-level questions to stimulate reason more than memory. He also suggests assigning an observer, who will ensure analysis and reflection on patient safety, communication, teamwork and leadership, among others^(^ 34 ^)^. The reflection of the group around the whole process carried out will be the end of the laboratory^(^ 15 ^,^ 34 ^)^.

Another study concluded that simulation as a pedagogical method allows students to recognize, interpret and integrate new information with previous knowledge in order to make decisions about the best direction to follow. The authors state that simulation, as an educational method, provides an opportunity to systematically structure learning to help students acquire deep content knowledge and to facilitate the development of thought processes; that simulation experiences stimulate students’ RACT skills and help them become more competent in caring for patients in complex conditions^(^ 37 ^)^.

We agree with what has been found in other studies emphasizing that simulation laboratories by themselves do not guarantee the development of RACT skills, but if combined with other strategies and implemented with adequate pedagogy, the results will be much more effective in terms of CT skills^(^ 34 ^,^ 37 ^-^ 38 ^)^.

It is also possible to find correspondence with that was discovered in the State of the Art of scientific production in RACT in the Andean region. The students perceive that “Clinical simulation is a valuable strategy for the acquisition, complementation and integration of the theoretical part with the practical part, because it seeks to make decisions according to the CT”^(^ 38 ^)^.

The evaluation of the subjects is cumulative and formative. In some cases, a diagnosis of the level of the student’s participation in the subject is made; it is evaluated in the intermediate and at the end with the objective of promotion to another level. In other cases, a teaching-learning balance is done to verify the fulfillment of the objectives and competences. Self-evaluation and heteroevaluation are increasingly used, implying a process of reflection, analysis and self-criticism.

Precisely, evaluation appears as one of the weakest points when analyzing the presence of RACT in curricula. Therefore, we agree that the “best teaching practice begins by establishing learning outcomes and continues with a focus on helping the student to achieve satisfactory results”. If the proposal is to achieve a higher order thinking, the evaluation will be oriented towards the synthesis, analysis and evaluation of knowledge^(^ 40 ^)^.

Overall, the strong approaches to RACT training formulated at HEI, HEIN, as evidenced by some of the teaching and learning strategies presented in the subjects, become much weaker in the evaluation process, with predominance of traditional evaluation models, and in some cases, the intention to evaluate RACT is outlined.

## Conclusion

The curricula of Colleges and Schools of Nursing in the Andean region explicitly contemplate reflective and critical thinking in their mission, vision, objectives, graduation profile and didactic strategies, and implicitly as integral formation. However, there is a tension between what is proposed by the HEI and HEIN and what is implemented and evaluated in the subjects. The presence of RACT in the proposed didactic strategies is much more evident, but it is not sufficiently objective or explicit in the evaluation processes.

Despite the great diversity of pedagogical models, there is a clear intention to facilitate the development of RACT. In addition, although a constructivist model is proposed centered on the student, dialogical, active, reflexive, innovative and critical, this model is more centered on the teacher than on the student; on knowledge over a relationship between equals; more on results than on the learning process. Likewise, knowledge is considered as something finished, fixed and the ultimate truth.

In order to be able to teach the RACT to the nursing student it is necessary to include it in the nursing curriculum, teachers who are professionals in the areas of Education and Nursing and with RACT in their training. Teachers should create spaces for the development of RACT, know and implement the different and complementary didactic strategies that facilitate its learning and that analyze the students in relation to the level of RACT achieved.

The authors of this article suggest that the projects currently developed with teachers and students in Ibero-America should be finalized and retaken with the implementation and evaluation of strategies that value the development of RACT.

RACT is considered an indispensable element in personal and professional development, in order to have autonomy, confidence, the ability to make decisions, reach clinical judgment and, the most important, provide individualized, comprehensive and human nursing care. In summary, graduates should be able to work as members of the health team with sufficient clarity of the role and identity they should have, because they have to integrate and experience the four paradigms of the Nursing.

### The limitations of the study are

The complexity of the project due to the number of participating countries and the different research groups;

The large number of public and private nursing schools and colleges in the Andean region;

The limitation in accessibility to the complete information of the curricula of each institution;

The minimal presence of information on the official web pages of each institution, school or nursing college;

No response and lack of interest from different schools and nursing colleges, public and private, to participate of this project;

Limited access of current and recent updates of the curricula of nursing colleges to develop this project.

The research group made efforts to reduce these limitations and devised multiple options that were proposed to the institutions, in order to facilitate the provision of information and its complementation when necessary.

### Applications for practice

The innovation and contributions expected with this research are based fundamentally on documenting and analyzing of the diverse existing evidences about if RACT is contemplated in the nursing curricula or not, the strategies used by teachers to create and promote it in nursing students and the evaluation processes employed. It provides insights about how RACT’s competence in nursing is addressed in the context of the Andean region and other regions of Ibero-America, its weaknesses and strengths, as well as the improvements that can be made. The final intention of the research is to offer, as a network and collegial body, proposals for teaching, learning and evaluation that will enable the empowerment of new generations of nurses, using RACT as a center of innovation and development.
